# An Idiopathic Sterile Thyroid Abscess in a Five-Year-Old Child

**DOI:** 10.7759/cureus.62257

**Published:** 2024-06-12

**Authors:** Asim Mehmood, Rida Inam, Zeshan Ali Siddiqui, Rehan E Kibria

**Affiliations:** 1 Internal Medicine, Shifa College of Medicine, Islamabad, PAK; 2 Internal Medicine, Shifa International Hospital, Islamabad, PAK; 3 Pediatric Surgery, Shifa International Hospital, Islamabad, PAK

**Keywords:** sterile, abscess aspiration, ultrasonography, idiopathic, thyroid abscess

## Abstract

We would like to share a case study of a five-year-old child who complained of a painful mass on the front of his neck, with a fever of 98°F. Sometimes thyroid abscesses may be overlooked due to their rarity and are lower down on our list of differential diagnoses. Neck ultrasonography confirmed our findings. A thorough analysis of the literature showed that causal bacteria are required for almost all abscesses. However, in our instance, the aspirated fluid culture revealed no growth, indicating that the abscess was sterile. When patients present with severe neck swelling, this case report emphasizes the significance of considering thyroid abscess as one of the differential diagnoses since early detection is critical to minimize the risk of complications.

## Introduction

Localized neck swelling in children has multiple causes, such as infective, developmental, inflammatory, and neoplastic, including benign and malignant tumors. Symptoms that follow abrupt swelling with pain and tenderness in the thyroid gland include fever, scratchy throat, dysphagia, hoarseness, and restricted head mobility [1]. In 1857, Bauchet published one of the first reports of an acute inflammatory thyroid disease. An isolated thyroid abscess in early childhood is an uncommon disease. Roughly 0.7% to 1% of thyroid disorders are caused by acute suppurative thyroiditis or thyroid abscesses. The thyroid gland's well-enveloped capsule, plentiful blood supply, and high iodine content usually render it immune to infections [2]. Abscesses of the thyroid are rare in contemporary medicine. In immunocompromised patients, those with pre-existing thyroid disorders, and with congenital abnormalities like pyriform sinus fistula and thyroglossal duct remnant, are often the ones who encounter them [3].

The patient usually presents with a painful swelling in the neck and fever, which can lead to a number of differential diagnoses, such as de Quervain thyroiditis, thyroid abscess, and fungal or tuberculous infection of the thyroid gland. Additional potential causes include lymphadenitis in the thyroid region, an infected thyroid tumor, and an infected branchial cleft cyst or sinus. Clinical differentiation between these entities may prove to be difficult, thus imaging and culture are crucial [4]. A complete blood count (CBC), a purified protein derivative test for tuberculosis, and titers for Epstein-Barr virus, cat scratch disease, cytomegalovirus, human immunodeficiency virus, and toxoplasmosis may be conducted as part of a proper workup to determine a final diagnosis, provided that the patient's medical history suggests any of these conditions. The best imaging method for finding a growing or palpable mass is ultrasonography. Computed tomography with intravenous contrast material should be used to investigate any suspected cancer or retropharyngeal or deep neck abscess [5].

## Case presentation

A five-year-old boy with no known comorbidities presented to the outpatient department with sudden onset of a tender swelling in front of the neck for five days, which had been gradually increasing in size, associated with moderate-grade fever, night sweats, and anorexia for five days; fever gets relieved after taking paracetamol. The patient did not report any recent upper respiratory tract symptoms of sore throat or cough. The patient complained of lethargy for a week. His medical and surgical history is insignificant, with no known drug or food allergies. On examination, vitals were in the normal range except for fever, which was recorded to be 99.8°F. Signs of anemia were evident in his conjunctiva. On inspection, a large swelling was obvious at the level of the superior pole of the left lobe of the thyroid. On palpation, it was warm, tender, firm in consistency, moving with swallowing. Moreover, submandibular, anterior, and posterior cervical lymph nodes were palpable.

Different laboratory investigations were ordered. The CBC showed a white blood cell count of 16130 (normal range: 4000-12000/microliter), a hemoglobin level of 10.2 (normal range: 11.5-14.5 g/dL), mean corpuscular volume (MCV) of 60.5 (normal range: 76-90 fL), and increased platelets at 496000 (normal range: 150,000-400,0000). Neutrophils were increased to 70% (normal range: 30-50%), while lymphocytes were reduced to 18% (normal range: 40-60%). Monocytes and eosinophils were also increased to 7% (normal range: 1-4%) and 5% (normal range: 1-2%), respectively.

**Figure 1:**
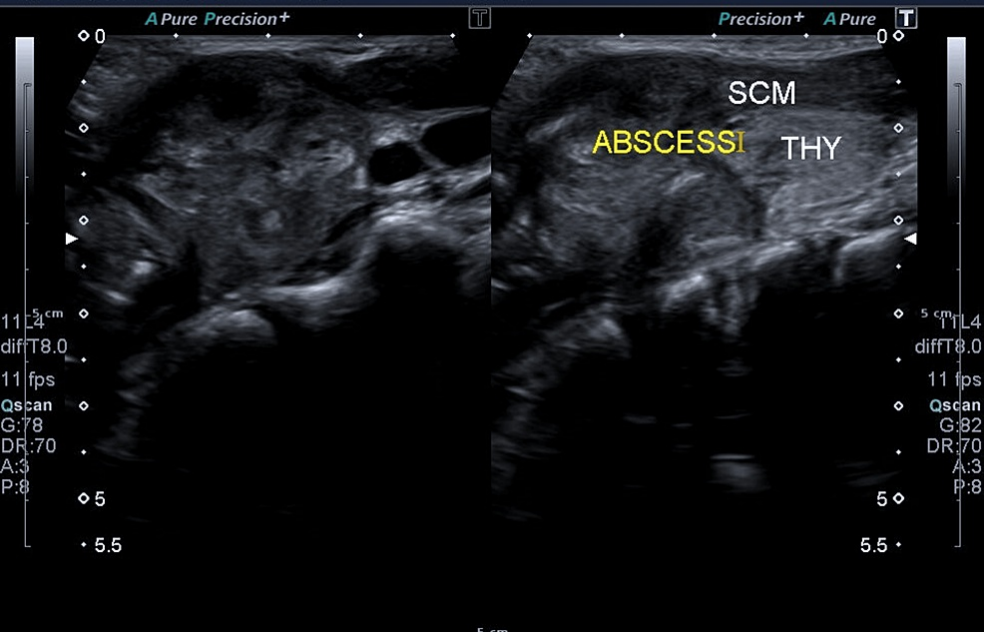
This ultrasonography image of the neck shows a large, vascularized area in the left thyroid lobe, potentially indicating an abscess, along with enlarged cervical lymph nodes nearby. SCM: sternocleidomastoid, THY: thyroid

Ultrasonography of the neck revealed a large heterogeneous area with internal vascularity measuring 24×24×29 mm (volume: 9 mL) at the level of the superior pole of the left lobe of the thyroid, with upper extension into the left sternocleidomastoid muscle, suggestive of abscess (Figure 1). Multiple enlarged and visible submandibular and posterior cervical lymph nodes with thickened hila were also observed. In the meanwhile, the patient was prescribed paracetamol syrup three times a day, and aspiration of the abscess was suggested.

Ultrasound-guided aspiration of the thyroid abscess was performed under local anesthesia and sterile conditions, yielding approximately 0.5 mL of pus. The remaining inflammatory phlegmon could not be aspirated due to its well-organized nature. Samples were subsequently sent for culture, gram stain, and acid-fast bacilli (AFB) stain. The culture was negative, and the gram stain revealed only a few pus cells, devoid of both gram-positive and gram-negative cocci and rods, as well as epithelial or yeast cells. Additionally, the AFB stain yielded negative results. The patient was given pediatric oral suspension of paracetamol, thrice a day for three days for his fever. In light of the absence of microbial growth in the culture, antibiotics were not prescribed. The recovery phase after aspiration proceeded without any complications. After the symptoms and swelling subsided, the patient was released the following day.

## Discussion

Thyroid abscess pathogenesis is not well understood. The thyroid gland, which has good blood flow, has a special defense against infection. Enclosed inside separated fascial planes, a capsule prevents infection propagation. More blood and lymph are given to the thyroid than to any other organ per gram of body weight. Other likely pathways of infection, excluding direct trauma, are internal fistulae from the pyriform sinus apex, lymphatic spread, hematogenous dissemination (via the superior, inferior, and thyroidea ima arteries), and a patent thyroglossal duct cyst [6]. Additionally, none of these prior issues were mentioned by our patient. While Staphylococcus, Streptococcus, and Pneumococcal species are the most often implicated organisms in abscesses, most infections are polymicrobial, and the flora varies significantly according to the patient's immunity. Evidence suggests that thyroid abscesses may be becoming more common in immunocompromised patients such as those with HIV infection [7]. Oropharyngeal flora was discovered in the remaining ones, including mixed growth, diphtheroid, alpha-hemolytic Streptococcus, and Neisseria species. The physical examination of the swelling, diagnostic imaging, and laboratory analysis are used to make the diagnosis of thyroid abscess in patients, who typically have a fever and a painful swelling in the front region of the neck [8]. 

Ultrasonography often reveals a heterogeneous thyroid gland echotexture with a superimposed anechoic or hypoechoic mass in cases of abscess. Although an infection can impact one or both of the thyroid gland's lobes, it often affects just one [9]. On the other hand, significant internal debris or bleeding associated with peripheral and interval vascular flow may cause an abscess' echotexture to alter. Under such conditions, ultrasonography alone might not be able to make a definitive diagnosis [10]. In our instance, the diagnosis might have been made with ultrasound alone, negating the need for other methods.

Antibiotics must be given (if a causative agent is identified) in addition to percutaneous or an incision and drainage treatment for thyroid abscesses. A common treatment for this condition is cephalosporins. In order to prevent consequences including retropharyngeal abscess, vocal cord paralysis, thyroid storm, and also suppurative mediastinitis, which can occasionally proceed to osteomyelitis or septic thrombophlebitis, thyroid abscesses need to be detected and treated as soon as feasible [11]. The abscess may burst and invade the esophagus or trachea if treatment is not received. Even with outpatient parenteral antibiotic therapy, surgical drainage and excision of any concomitant anatomic defects were necessary in several cases to decrease the risk of recurrence of the abscess [12].

## Conclusions

Although thyroid abscess is uncommon, physicians should take it into consideration when making a differential diagnosis for acute neck swelling. Early diagnosis is essential for starting the right treatment and lowering morbidity. This case highlights the importance of a thorough diagnostic strategy that includes staining and culture methods. Microbiological examinations are crucial for directing antibiotic therapy and averting potential problems. It is essential to collaborate across multiple disciplines in order to achieve positive patient outcomes and lower the likelihood of recurrent abscesses.
